# Learning Ordinal Embedding from Sets

**DOI:** 10.3390/e23080964

**Published:** 2021-07-27

**Authors:** Aïssatou Diallo, Johannes Fürnkranz

**Affiliations:** 1 Research Training Group AIPHES, Technische Universität Darmstadt, 64289 Darmstadt, Germany; diallo@ke.tu-darmstadt.de; 2 Computational Data Analytics, FAW, Johannes Kepler University Linz, 4040 Linz, Austria

**Keywords:** ordinal embedding, sets, representation learning

## Abstract

Ordinal embedding is the task of computing a meaningful multidimensional representation of objects, for which only qualitative constraints on their distance functions are known. In particular, we consider comparisons of the form “Which object from the pair (j,k) is more similar to object i?”. In this paper, we generalize this framework to the case where the ordinal constraints are not given at the level of individual points, but at the level of sets, and propose a distributional triplet embedding approach in a scalable learning framework. We show that the query complexity of our approach is on par with the single-item approach. Without having access to features of the items to be embedded, we show the applicability of our model on toy datasets for the task of reconstruction and demonstrate the validity of the obtained embeddings in experiments on synthetic and real-world datasets.

## 1. Introduction

The objective of an ordinal embedding algorithm is to find a low-dimensional Euclidean representation of a number of abstract items, for which no feature representation or numerical distance information is available. Instead, the learner has access to a set of comparisons where for a quadruple of points i,j,l, and *k* from an abstract space X, it is specified whether the pair (i,j) is closer to each other than the pair (l,k), i.e., whether δ(i,j)<δ(l,k) for some latent distance function δ [1,2]. A special case of this problem results when points *i* and *l* coincide, i.e., when the learner has access to triplet comparisons, which specify for three objects *i*, *j*, and *k* whether *i* is closer to *j* or to *k*.

Several tasks in Machine Learning (ML) and Information Retrieval (IR) depend on some underlying notion of similarity between objects. Supervised learning attempts to assign labels to objects based on the notion of feature similarity, while unsupervised learning attempts to discover hidden patterns of similarity between objects. Prior work in this area has focused on various aspects of the ordinal embedding problem. Agarwal et al. [3] provided a flexible and modular algorithm with proven convergence guarantees. Later work focused on explaining disagreement among human assessors, modeled by noisy triplets, and more tailored to crowd-sourcing [4,5,6]. Terada and von Luxburg [7] promised embeddings that recovered the exact point position with application for density estimation. Other works focused on theoretical aspects of the ordinal embedding problem. For example, Kleindessner and von Luxburg [8] proved that under reasonable distributional assumptions, it is possible to recover an embedding that places all objects within a reasonable error range of their correct position, and Jamieson and Nowak [9] showed that the triplet selection phase is as critical as the algorithm itself and derived a lower bound on the number of triples necessary for recovering an ordinal embedding. In our own prior work [10], we proposed a method for finding distributional ordinal embeddings, i.e., embeddings that can also model and explicitly represent the uncertainty of the location of a point recovered from noisy comparisons.

A common application for ordinal embedding methods is crowd-sourcing. In practice, the triplet comparisons are often obtained by combining the answers from multiple human assessors that are asked to give subjective feedback. Moreover, it has been shown that eliciting such ordinal feedback is more reliable than the feedback based on the question “how close is item *i* to item *j*” [11]. The unequivocal advantage is that this method is a solution for the issue of comparing subjective scales across different crowd-workers.

In this work, we extend this concept to the case when the learner is not presented with triplets of abstract items, but rather, sets of abstract items. We are now given (unordered) sets of items and training triples that provide the form “the set of items *J* is closer to the sets of items *I* than the set of items *K*”, where I,J,K⊂X. Note that the sets may have overlaps, so that each element x∈X may occur in multiple sets. The task is now to output a meaningful representation of all items x∈X in a low-dimensional space that respects the observed constraints. Obviously, this problem is a generalization of the classical ordinal embedding problem, where each set only consists of a single element. The set-based formulation is particularly useful when the number of abstract items is very large and/or the number of times the oracle that yields the training information can be interrogated is limited. How can we build a model that deals with sets of abstract values and outputs sets of low-dimensional representation while satisfying the triplet constraints? This paper aims at answering this question. We summarize our main contributions as follows:

- A Set-valued Ordinal Embedding (SetOE) is proposed to embed data points in a low-dimensional space. We reformulate the classical ordinal embedding problem based on single data points into a generalization based on sets while assuring the permutation invariance necessary when dealing with the set. We develop an architecture to allow for conditioning with possibly different sizes of sets and adapt the margin-based loss for set-valued input;
- We propose a distributional approach that does not rely on the features of the individual data points for the ordinal embedding problem with sets. We motivate the advantages of such a setting and explain the properties we use to enable this;
- Experiments on both artificial and reals datasets demonstrate the validity of our approach for embedding datasets of considerable size in a significantly low-dimensional space (e.g., two). We evaluate our approach on several datasets. First, we present a proof-of-concept with different synthetic datasets. Then, we escalate the complexity of the tasks to the MNIST dataset, poker, and more real-word datasets such as Reuters.

The remainder of the paper is organized as follows: In Section 2, we formally introduce the ordinal embedding problem and lay down the mathematical preliminaries, as well as the notation used throughout the paper. In Section 3, we present our approach for a set-valued ordinal embedding, which we evaluate in Section 4 with empirical studies in a variety of datasets. Finally, Section 5 collects related work on ordinal embedding, set-valued input, and representation learning, before we draw some conclusions in Section 6.

## 2. Ordinal Embedding

In this section, we formally state the ordinal embedding problem and establish the notation, for which we follow [12]. ∥·∥ denotes the $\ell_{2}$ norm. $S_{+}^{d}$ is the set of all positive-definite matrices. In the scope of this work, we only focus on Gaussian distributions, which belong to the family of parametrized probability distributions $z_{h,a,A}$ having a location vector $a\in R^{d}$, which represents the shift of the distribution, a scale parameter $A\in S_{+}^{d}$, which represents the statistical dispersion of the distribution, and a characteristic generator function *h*. Specifically, for Gaussian distributions, the scale parameter coincides with the covariance matrix $var(z_{h,a,A})=A$. From now on, we denote Gaussian distributions (or embeddings) as $z_{\left(h,a,A\right)}=N\left(a,A\right)$.

### 2.1. Classical Ordinal Embedding

The ordinal embedding problem, also called nonmetric multidimensional scaling [1,2], aims at obtaining the corresponding embeddings in a low-dimensional space. Consider *n* items in an abstract space X, which, without loss of generality, we represent by their indices [n]=1,⋯,n. It is worth mentioning that no explicit representation of the items is available, so it is not possible to analytically express the dissimilarity between the items. We thus assume a latent underlying dissimilarity (or similarity) function $\delta :X\times X\overset{}{\to }R_{\geq 0}$, which cannot be directly observed, but information on δ is indirectly available via a set of training triples.

Let T:={〈i,j,k〉:1≤i≠j≠k≤n} be a set of unique triplets of elements in X. We further assume an oracle O, which provides a binary label +1 or −1 for each triplet 〈i,j,k〉∈T, indicating whether δ(i,j)<δ(i,k) holds or not:

(1) $$O\left(\langle i,j,k\rangle \right)=\left\{\begin{array}{cc} +1 & \mathrm{if}\;\delta (i,j)<\delta (i,k)\\ -1 & \mathrm{if}\;\delta (i,j)>\delta (i,k) \end{array}\right.$$

Note that at this stage, we do not require δ to be a metric. Together, T and O represent the observed ordinal constraints on distances.

The learning problem problem can now be formally defined as follows:

**Definition** **1** (Ordinal embedding)**.** *Given n points {1,⋯,n} in an abstract space X, a set of triplets $T\subset X^{3}$, and an oracle $O:X^{3}\to \left\{-1,1\right\}$, which provides information about a latent similarity function δ as specified in* (1)*, the* ordinal embedding problem *consists of finding a suitable embedding function $\phi :X\overset{}{\to }R^{d}$, such that:*

(2) sgn(∥ϕ(i)−ϕ(k)∥−∥ϕ(i)−ϕ(j)∥)=O(〈i,j,k〉)

### 2.2. Distributional Ordinal Embedding

An extension to the classical ordinal embedding problem is to learn probabilistic embeddings in lieu of the conventional Euclidean embeddings, taking advantage of the fact that vectors can be considered as an extreme case of probability measures, namely Dirac [12]. For this purpose, we focus on the family of elliptical distributions, more precisely Gaussian distributions, which enjoy many advantages. The main results were extracted from our previous work [10], which extended the ordinal embedding problem defined in Section 2.1 from Euclidean embeddings to Gaussian embeddings.

Hence, the considered problem becomes:

**Definition** **2** (Probabilistic ordinal embedding)**.** *Suppose $T\subset X^{3}$ is a set of triplets over X and $O:X^{3}\to \left\{-1,1\right\}$ is an oracle as defined in* (1). *Let $Z=\{z_{1},\ldots ,z_{n}\}$ be the desired probabilistic embedding, where each of the original points $x_{i}$ is mapped to the probability distribution parameterized by $z_{i}$ and Δ(.,.), a distance function between distributions.* Probabilistic ordinal embedding* is the problem of finding a function $\psi :X\overset{}{\to }Z$ that maps each point i∈X to a probability distribution $z_{i}=\psi \left(i\right)$, such that:*

(3) $$\mathrm{sgn}\left(\Delta \left(z_{i},z_{j}\right)-\Delta \left(z_{i},z_{k}\right)\right)=O\left(\langle i,j,k\rangle \right),$$

*for 〈i,j,k〉∈T.*

This definition requires a distance measure Δ between distributions. For this purpose, we selected the *Wasserstein distance* [13], also known as the *Earth mover’s distance*, which has been previously used as a loss function for supervised learning [14] and in several applications.

#### 2.2.1. The Two-Wasserstein Distance

In Optimal Transport (OT) theory, the Wasserstein or Kantorovich–Rubinstein metric is a distance function defined between probability distributions (measures) on a given metric space *M*. The squared Wasserstein metric for two arbitrary probability measures $\mu ,\nu \in P(R^{d})$ is defined as:

$$W_{2}^{2}\left(\mu ,\nu \right)\overset{\mathrm{def}}{=}\underset{X∼\mu ,Y∼\nu }{\inf}E_{{∥X-Y∥}^{2}}$$

In the general case, it is difficult to find analytical solutions for the Wasserstein distance. However, a closed-form solution exists in the case of Gaussian distributions. Let $\alpha \overset{\mathrm{def}}{=}N(a,A)$ and $\beta \overset{\mathrm{def}}{=}N(b,B)$, where $a,b\in R^{d}$ and $A,B\in S_{+}^{d}$ are positive semi-definite. Hence:

(4) $$W_{2}^{2}\left(\alpha ,\beta \right)={∥a-b∥}^{2}+B^{2}\left(A,B\right)$$

where $B^{2}$ is the *squared Bures metric* [15], defined as:

(5) $$B^{2}\left(A,B\right)\overset{\mathrm{def}}{=}\mathrm{Tr}\left(A+B-2{\left(A^{\frac{1}{2}}BA^{\frac{1}{2}}\right)}^{\frac{1}{2}}\right)$$

When $A=\mathrm{diag}\;d_{A}$ and $B=\mathrm{diag}\;d_{B}$ are diagonal, $W_{2}^{2}$ simplifies to the sum of two terms:

(6) $$W_{2}^{2}\left(\alpha ,\beta \right)={∥a-b∥}^{2}+h^{2}\left(d_{A},d_{B}\right)$$

where $h^{2}\left(d_{A},d_{B}\right)\overset{\mathrm{def}}{=}{∥\sqrt{d_{A}}-\sqrt{d_{B}}∥}^{2}$ is the *squared Hellinger distance* [16] between the diagonal $d_{A}$ and $d_{B}$.

#### 2.2.2. Learning Gaussian Embeddings

As mentioned in Definition 2, our goal is to learn a function that maps each item *i* to a parametrized probability distribution $z_{i}$, such that the two-Wasserstein distances between the embeddings satisfy as many triplets as possible. In our case, we use a *d*-dimensional Gaussian embedding, so that $z_{i}=\left(\mu_{i},\Sigma_{i}\right)$. Let $E_{ij}$ be the energy function between two items (i,j) [17], which characterizes our energy-based learning approach. In particular, we set $E_{ij}=W_{2}^{2}\left(z_{i},z_{j}\right)$. Finally, the corresponding optimization problem is the following:

(7) $$\max_{z_{1},\cdots z_{n}\in R^{d}}\sum_{t=(i,j,k)\in T}O\left(t\right)\cdot \mathrm{sgn}\left(E_{ij}-E_{ik}\right)$$

which is discrete, nonconvex, and NP-hard. For these reasons, a relaxation of this optimization problem is needed. We make the choice of using the hinge loss L((t=〈i,j,k〉,O(t)), a well-established loss function in contrastive metric learning, as a convex surrogate:

(8) $$L=\sum_{t=\langle i,j,k\rangle \in T}\max\left(1-O\left(t\right)\cdot \left(E_{ij}-E_{ik}\right),0\right)$$

The empirical performance of embedding methods is evaluated by the *empirical error*, also called the *triplet error*.

(9) $$Err=\frac{1}{|T^{\prime }|}\sum_{\langle i,j,k\rangle \in T}𝟙\left[\left(y\cdot \mathrm{sgn}\left(E_{ij}-E_{ik}\right)\right)=1\right]$$

## 3. Ordinal Embedding for Sets

This section contains our primary contribution: an approach for encoding sets of abstract items into sets of low-dimensional vectors. As previously established for the ordinal embedding problem, the only supervision given is in the form of triplet comparisons by an oracle O(t) that takes as the input the triplet of sets t=〈I,J,K〉 and returns a value {−1,+1}.

### 3.1. Problem Statement

As in conventional ordinal embedding, we consider *n* items in the abstract space X, which we represent without loss of generality by their indices [n]=1,⋯,n. Without loss of generality, we represent a set *X* as an unordered collection of indices of size $k_{X}$, i.e., $X=\{x_{1},\cdots ,x_{k_{X}}\}\subset X$.

The input to our model is a triplet of sets t=〈I,J,K〉. The sets can be overlapping, so that each item i∈X may occur in arbitrarily many sets. Furthermore, each set *X* can have different cardinalities $k_{X}$, so a constant set size is not a prerequisite of our approach. However, in the following, if the context is clear, we omit the set index and denote the cardinality of each set as *k*. Analogous to (1), we assume that each triplet in the set of training triplets T has been labeled by an oracle O as:

(10) $$O\left(\langle I,J,K\rangle \right)=\left\{\begin{array}{cc} +1 & \mathrm{if}\;\delta (I,J)<\delta (I,K)\\ -1 & \mathrm{if}\;\delta (I,J)>\delta (I,K) \end{array}\right.$$

where δ(.,.) is a latent, unspecified similarity function between sets.

The goal is to learn a mapping function that takes as the input a set of indices X and a training set of labeled triplets T defined over elements in X and outputs a set of vectors in $R^{d}$ corresponding to the embedding of the individual items that compose X. Formally, we can define the problem as follows:

**Definition** **3** (Set-based ordinal embedding)**.** *Let X be an abstract space of items, which we denote with {1,⋯,n}, and $P_{X}=2^{X}\times 2^{X}\times 2^{X}$ the space of all triples of subsets of X. Given $T\subset P_{X}$ and an oracle $O:P_{X}\to \left\{-1,1\right\}$, which provides information about a latent similarity function δ as specified in* (10)*, the* set-based ordinal embedding problem *consists of finding a suitable embedding function $\phi :X\overset{}{\to }R^{d}$, such that:*

(11) sgn(∥agg(Φ(I))−agg(Φ(K))∥−∥agg(Φ(I))−agg(Φ(J))∥)=O(〈I,J,K〉)

*where Φ(.) denotes the elementwise extension of ϕ(.) to sets and $\mathrm{agg}\left(.\right):R^{d\times k}\to R^{d}$ is an aggregation operator defined over elements in $R^{d}$.*

### 3.2. Set Encoding

Clearly, the ordinal embedding for sets problem is a natural generalization of the classical ordinal embedding problem as defined in Definition 1: The generic input for our model is not a single item, but a set of items $X=\{x_{1},\cdots ,x_{k}\}$ of size *k*, where each $x_{i}$ is an indexed item of X. The output of the model is a set of feature vectors of dimensionality *d* represented by the matrix $Y=\Phi \left(X\right)\in R^{d\times k}$ with the column elements $Y=[y_{1},\cdots ,y_{k}]$, where Φ is the learned mapping function.

In order to properly deal with sets, all the operations that compose Φ need to have the properties of permutation equivariance and permutation invariance. That is to say that Φ should not rely on the arbitrary order of the elements of the input set. The approach proposed in this paper relies on set encoders. A *set encoder* is a model that encodes a set of elements into feature vectors in a latent space. They are built as a composition of permutation-equivariant operations with a permutation-invariant operation at the end. Specifically, we assume that the function Φ can be decomposed into an elementwise function ϕ(.), which can be independently applied to each element, i.e.,

(12) $$\Phi \left(X\right)=\left[\phi \left(x_{1}\right),\cdots ,\phi \left(x_{k}\right)\right]=\left[y_{1},\cdots ,y_{k}\right].$$

Essentially, the function ϕ(.) corresponds to the elementwise function of Definition 1. The resulting Φ(.) is permutation equivariant because its defining function ϕ(.) is applied to every element individually. Hence, it does not rely on the arbitrary order of the input set, and the the order of the output will adapt to any change in the order of the input.

Since the supervision is available only for the set and not for the single components, intuitively, the vectors ${\left\{y_{i}\right\}}_{i=1}^{k}$ need to be aggregated into a single vector using an aggregation function agg(.). Obviously, agg(.) also needs to respect the property of permutation invariance. Multiple operations abide by this rule, for example the sum, average, min, or max. This allows us to obtain a representation of the set and its elements regardless of the order in which of the set elements are presented. In our experiments, we focus on the *centroid*, the center of mass $\bar{y}\in R^{d}$, i.e.,

(13) $$\mathrm{agg}\left(Y\right)\overset{[0pt]\mathrm{def}}{=}\bar{y}=\frac{1}{k}\sum_{k}y_{k}.$$

### 3.3. Distributional Embeddings for Sets

Learning ordinal embeddings from triplet comparisons based on sets is a problem that can lead to substantial approximations and imprecisions. One of the main advantages of the distributional approach outlined in Section 2.2 is that it is possible to represent and address severe perturbations in the data. As stated earlier, the representation through probability measures naturally allows encapsulating the uncertainty about the representation. Hence, following this approach, we further generalize our approach to a distributional embedding, where each set in the target space corresponds to a probability distribution.

To that end, we chose the Gaussian distribution N(μ,Σ) characterized by a location vector μ and a covariance matrix Σ, i.e., we associated each set X with a distribution $z_{X}=\left(\mu_{X},\Sigma_{X}\right)$, where $\mu_{X}=\bar{y}$, i.e., the centroid (13) of the set, and, following what is detailed in Section 2.2, a diagonal covariance matrix $\Sigma_{X}$. Overall, such a distributional embedding for sets allows obtaining a meaningful representation for each set, while being bounded by the components of the sets themselves.

The updates performed on a single training triplet are illustrated in Algorithm 1. Essentially, it takes a set triplet 〈I,J,K〉 and updates the item embeddings for all the elements in these sets, so that the hinge loss (8), which is based on the Wasserstein distances between the distribution of the item embeddings of the elements in each of the three sets, is reduced.
**Algorithm 1** Distributional ordinal embeddings from set constraints.**Require:** set of items X, set of training triplets T, oracle O**Ensure:** set embeddings Y of points in X 1:initialize Y randomly 2:**for all**(t=〈I,J,K〉)∈T**do** 3: **for all** (S∈{I,J,K} **do** 4:  $Y_{S}=\left\{y_{1},\cdots ,y_{\left|S\right|}\right\}$ column vectors of Y corresponding to *S* 5:  $z_{S}\leftarrow N\left(\mu_{S},\Sigma_{S}\right)$                {mean and variance of $Y_{S}$} 6: **end for** 7: $l\leftarrow \max(1-O\left(t\right)\left(W\left(z_{I},z_{J}\right)-W\left(z_{I},z_{K}\right)\right),0)$   {compute hinge loss} 8: $Y_{\cdot }\leftarrow Y_{\cdot }-\eta \frac{\partial l}{\partial Y}$                   {gradient descent step} 9:**end for**

### 3.4. Deep Set Encoder

**Figure 1:**
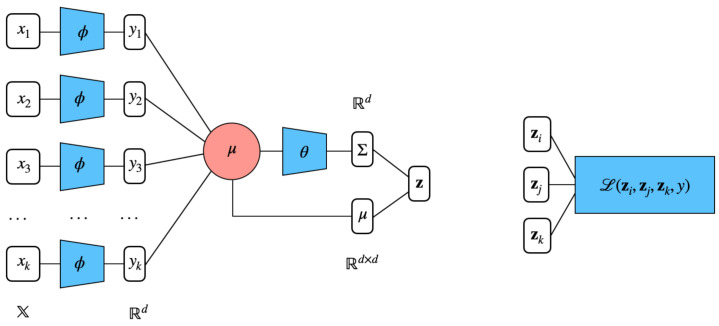
Distributional ordinal embeddings for sets. Each element of the input set *i* is fed to identical 2-layer MLP and outputs $y_{i}$. These feature representations are averaged into μ, the centroid of the set. Finally, θ takes as input μ and outputs the covariance matrix Σ (in our case, it is reduced to the diagonal of the covariance matrix). Coupled with the previously obtained μ, these vectors constitute the distributional embedding of a given input set. The Wasserstein-based hinge loss allows the optimization for learning the low-dimensional representation of the elements of a triplet of sets.

We represent the elementwise embedding function ϕ(.) as a deep neural network, as illustrated in Figure 1. While our work relates to numerous architectures proposed in metric learning such as that in [18], our deep neural encoder is fundamentally different because of the nature of the problem. The most distinctive point is that we do not have access to the features of the items we aim to embed. In fact, our model learns a representation of the items based on a random input to the encoder. In particular, we chose as inputs random vectors on input dimension h=64 sampled from $N(0,I_{h})$. A first deep encoder, namely two-layer MLP with ReLU, $\phi_{\theta }\left(\cdot \right)$ maps these random inputs into *d*-dimensional outputs. These are then aggregated to $\mu_{\theta }\left(\cdot \right)$ and fed to produce the variance $\Sigma_{\theta }\left(\cdot \right)$, a function, which is again represented with a deep forward network.

### 3.5. Complexity

The training complexity is linear in the size of T, which is the set of all triplets, and bounded by $O(n^{3})$. However, a well-chosen sampling strategy may decrease this bound. It has been shown by Jamieson and Nowak [9] that the minimum number of triplets to recover an ordinal embedding is Ω(ndlogn) in $R^{d}$. We adapted this result to the setting in which the parameters to be learned are a mean vector in $R^{d}$ and a covariance matrix $S_{+}^{d}$. Hence, the dimensionality can be considered to be $d^{\prime }=d+d^{2}$ and $O(d^{2})$. Thus, the newly recovered lower bound for the triplets becomes $\Omega (d^{2}n\log n)$, which is still polynomial in *d* and O(nlogn). Since ordinal embeddings typically map into a low-dimensional space, this is not a drastic loss in efficiency. Moreover, it is worth mentioning that a low number of epochs was needed for convergence for all experiments. Finally, the computational bottleneck when dealing with Wasserstein distance in its closed-form is computing the matrix square roots of the scale parameters. However, as we opted to learn diagonal covariances, hence this problem is not present in our approach.

### 3.6. Practical Tricks

Besides relying on the optimization of the energy-based max-margin loss (8), we applied some regularization to the learning process. We observed that no regularization is needed for learning the location vectors. However, the covariance matrix needs to be bounded, since the main goal of our approach is to obtain perceptual embeddings. Hence, we constrained the covariance matrix to lie within the hypercube ${\left[0,C\right]}^{d}$, *C* being a chosen constant. We chose to focus on diagonal covariance because we argue the rotation angle is not easily interpretable to appreciate the similarity between items and that the principal axes are sufficient to appreciate the uncertainty of the representation. Thus, the regularization is achieved by bounding each element of the covariance matrix, $\Sigma_{ii}=\max\left(\Sigma_{ii},C\right)$. Moreover, we adapted our approach to be able to handle sets of variable size. First, we padded all sets in a batch to allow for efficient computation. We then provided an additional mask feature $m_{i}$ for each set $P_{i}$ that indicates whether it is a regular element of the set $\left(m_{i}\left(k\right)=1\right)$ or padding element $\left(m_{i}\left(k\right)=0\right)$. This mask is useful for computing the centroid of the set through the weighted average and the covariance of the set.

## 4. Experiments

Our main objective was to investigate the effectiveness of our approach for the ordinal embedding problem. With this objective in mind, we evaluated our model in two different settings. First, we performed experiments with reconstruction tasks on synthetic datasets with particular shapes. These sets of experiments were particularly useful as a controlled environment because the ground truth was available. Then, we applied our approach to real-world datasets for more complex data in order to assess the performance of our model in real cases.

In all experiments, we used the following hyperparameters: $l_{r}=1e-3$ as the learning rate, batch size 512, h=64 as the hidden size of the input layer, and d=2, the output size of the embeddings. We compared our proposed model to a model that learns to embed a set simply as its centroid. In order to evaluate our approach, we used as the metric the Procrustes distance, as well as the triplet error (9) between the ground truth and learned embeddings.

### 4.1. Synthetic Datasets

**Figure 2:**
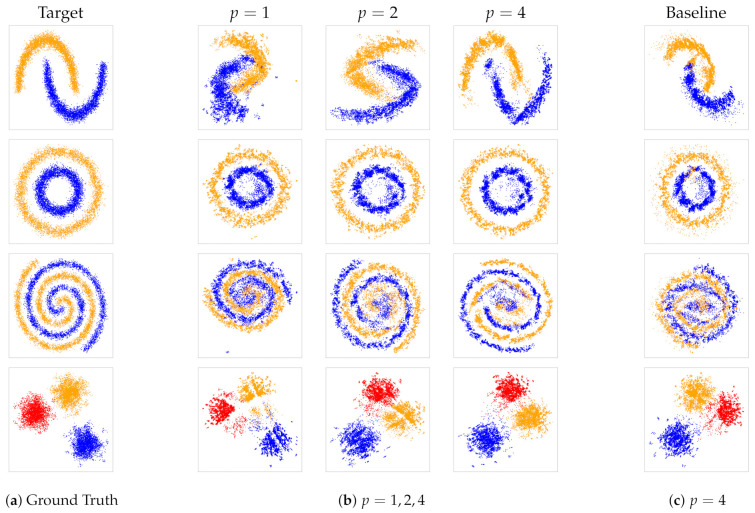
SteOE embeddings for the synthetic experiments. The first column (**a**) shows the ground truth, (**b**) the progression of learned embeddings from increasing number for triplets pndlogn with p={1,2,4}, and (**c**) the learned embedding from the baseline model with p=4. The colors are merely used for better visibility of the different groups; they were not used for training.

**Data.** In this section, we present a series of experiments that use reconstruction tasks in order to illustrate the capabilities of our approach. We followed the experimental setting of [19]. More specifically, we used 4 2-dimensional synthetic datasets generated with the scikit-learn package in Python. The datasets were:

(i) Gaussian isotropic blobs;
(ii) A large circle containing a smaller circle in 2D;
(iii) Two interwoven spirals;
(iv) Two interleaving half circles;

as illustrated in Figure 2a. For each of these datasets, we proceeded as follows:

- Fix *n* and $k_{i}$ with i=1,⋯,n, respectively the number of sets of items, and the size of each set;
- Generate *n* points $c_{i}$ that follow the pattern of the chosen dataset. These points are the centroids of the *n* sets;
- Given $c_{i}$, draw $k_{i}$ random points from a normal distribution parameterized as $N(c_{i},\epsilon )$. We chose ϵ, the spread of the set, between 0 and 0.5;
- We divided the obtained *n* cloud of points in overlapping sets.

Following the approach described, we generated |T| triplets sampled from a uniform distribution. In order to simulate the ordinal feedback from the oracle, we computed the difference of the squared $l^{2}$ norm between the centroids of the sets for a given triplet. The total number |T| of sets was set to be pndlogn with p=1,2,4.

**Results.** This series of experiments on synthetic datasets illustrates the performance for reconstruction and density estimation of our approach and, in particular, the influence of the number of triplets on the reconstruction ability. The first column of Figure 2 depicts the ground truth. Then, proceeding left to right, the embeddings werelearned for different values of *T*. The number of triplets increases with T=pndlogn, where p={1,2,4}.

For all datasets, we observed that the quality of the reconstruction with respect to the location of the point vector improves when the number of triplets increases. We recall that an ordinal embedding is not unique, but the distances can be recovered up to an orthogonal transformation (translation, rotation, and reflection).

**Figure 3:**
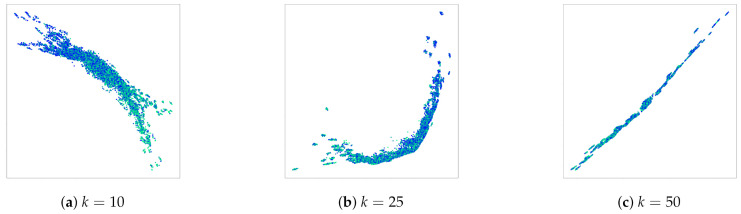
Ordinal embeddings of MNIST digits. The supervision information is the sum of the digits in the sets. Colors represent the label of single digits, used only for visualization purposes.

The last column in Figure 3c is the visualization of the embeddings obtained by the baseline model, which is visibly less accurate than the proposed model. In order to obtain a quantitative, objective evaluation of the difference, Table 1 shows the Procrustes distance between the ground truth and the resulting embedding for both our proposed model and the baseline approach. We notice that our embeddings are consistently more precise, and this suggests that our distributional approach for set embedding leads to a better and more accurate representation.

### 4.2. Sum of MNIST Digits

**Data.** Next, we applied our approach to more complex distributions than the synthetic datasets previously illustrated. We adapted the MNIST dataset for this task. MNIST contains 60,000 instances of 28×28 grey-scale stamps of digits in the range 0,⋯,9. We randomly sampled *N* = 100,000 for training and 1000 for testing with a maximum size of *k* = 10, 25, 50 images. The supervision information provided by the oracle (10) is based on the difference of the sum of the digits in a set, i.e.,

(14) $$\delta \left(I,J\right)=|\;\sum_{i\in I}\lambda_{i}-\sum_{j\in J}\;\lambda_{j}|$$

where $\lambda_{i}$ is the label of image *i*, i.e., the one-digit number displayed by it. Thus, we cannot directly observe the label of the image, but we can only observe whether it tends to appear in sets with larger or smaller sums. The desired outcome is that the embedding of the individual images is able to capture the hidden label information.

**Results.**Figure 3 illustrates the obtained results for three different maximal set sizes. Each point represents a single image of the MNIST dataset. The color indicates the label of each single digit. We can clearly recognize the linear order in the learned embeddings. Low digits are separated from high digits, and the gradient is clearly noticeable.

Moreover, the smaller the sets used for learning, the more the order is distinguishable. In fact, although it is clear that the model was able to capture the linear order from the feedback of each triplet comparison, the results of Figure 3c are not as clear as those of Figure 3a. This can be expected, because in larger sets, the contribution of each individual number of the sum is lower than in smaller sets. This is an important factor from which we conclude that there exists a trade-off between the reconstruction ability and precision of the obtained representations. When density estimation is the priority, a bigger set size is advantageous because it necessitates fewer comparisons. However, when the focus is on the preciseness of the location of individual items in the space, a smaller set size should be preferred. Finally, we train a simple classifier on the learned embeddings to whose objective is to predict the label of the single MNIST digits. The results are reported in Table 2 in terms of mean accuracy. We can notice that our embeddings perform better than the ones learned through the baseline model, which proves the validity of our approach.

### 4.3. Poker Hands

**Data.** Poker is one of the best-known card games. The players bet whether the value of the hand they hold will beat all others according to a predefined ranking of hands. The complexity of the ranking system, where each card can be a part of a winning hand depending on the other cards in the hand, provides an interesting use case for assessing the embedding abilities of our approach. Variants largely differ on how cards are dealt and the methods by which players can improve a hand. In our experiments, we modeled a setting that was motivated by the Texas hold’em variant. We assumed two players *J* and *K*, each holding *p* cards, and a set of *c* community cards *I*.

The supervision information we used was based on which of the two players could obtain the best hand of five cards by combining his/her own cards with the community cards. The hand strength computations were based on Cactus Kev’s algorithm.

**Figure 4:**
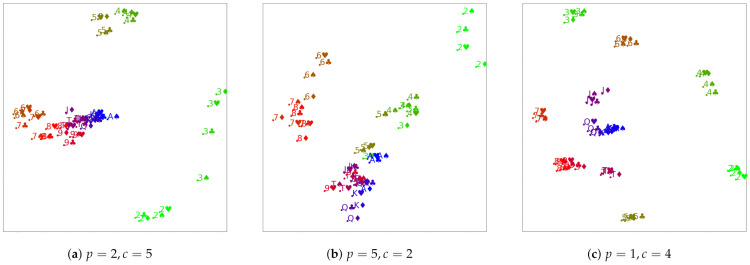
Ordinal embeddings from poker for different numbers of player cards *p* and community cards *c*.

**Results.**Figure 4 illustrates the results. In all cases, the number of triplets is 2ndlogn, with d=2 and n=52. The colors show the rank of the cards, but this information was not available during the training. We illustrate three different variants:

Figure 4a shows the results of the setting with five community cards and each of the two players having two cards. This corresponds to the Texas Hold’em game.

The remaining figures show variants with differing numbers of board and community cards, which do not correspond to actual game settings, but which we studied to gain more insight into the obtained embeddings.

The results for the classical variant (Figure 4a) show that even if the supervision comes from a highly nonlinear source, our approach is still able to learn ordinal embeddings and output latent representations for the game that are fair and interpretable. First, we notice that unlike in the previous experiment, where a clear linear order of the embedded MNIST images was obtained, the structure obtained here is more complex. Nevertheless, we see that cards with similar values tend to form clusters because they go well together, forming pairs, triples, or even pokers, which have a high evaluation in the game. We can also see that clusters of cards with a low rank are pushed afar, whereas cards with higher ranks tend to be closer to each other. This, again, makes sense from the perspective of the game, because two high cards (regardless of whether they match or not) are a good combination. In particular, aces, being the cards with the highest rank in the deck, remain in the center of the plot. This finding is in accord with the rules of the game, since the probability of having a good hand is higher if it includes aces. It is possible to notice how the clusters are arranged in a spiral-like shape with the aces being in the center and moving farther away, and we can find the other values in decreasing order.

We then chose to evaluate the opposite setting of the one just described, in which the board has less cards than each player hands, more specifically two cards for the board and five for each player. This corresponds to Figure 4b. Even though this setting does not correspond to any game configuration, we assumed that investigating it could be of interest. Once again, we reached the same conclusions: cards with similar ranks are close to each other, also arranged in a spiral-like shape emanating from the center of cluster of aces. However, contrary to the canonical setting, the cluster of aces is further from the middle of the point, and this is probably due to the higher combinatorial nature of the evaluation that perturbed the original order.

In the next setting, we tried to remove the combinatorial factor of the excess community cards. For this, we used the configuration in which each player had one card (a singleton) and the board had four cards. The results, shown in Figure 4c, exhibit the best separation between the different clusters of ranks and the spiral shape of their arrangement. Low ranks are far apart from high-ranked cards, and the higher ones are closer to the center, with the cluster of aces being the most centered one.

Overall, the found embeddings appear to be quite reasonable in all three cases. They seem to capture the expected property that cards that go well together in a poker hand have a low pairwise distance, whereas pairs of hands that do not go well together are further apart. For that reason, the low cards are rather far from all other cards except for their own kind, whereas the higher cards tend to be closer to each other. In order to test this, we computed a correlation coefficient between the distance of a pair of cards and the pairs’ Chen score (the Chen score is a formula proposed by Bill Chen for assessing the strength of a pair of starting cards in Texas hold’em [20]) for all pairs of cards. As expected, the results, in Table 3, show a reasonably high positive correlation.

### 4.4. Reuters

**Data.** For this experiment, we used data from the Reuters-21578 benchmark corpus [21]. This dataset contains *n* =10,788 Reuters Newswire articles. Each article is represented as a set of paragraphs with size k∈{2,15}, with 60,222 paragraphs in total. The goal was to cluster these documents according to the categories of the newspaper. The documents belong to 90 different categories. The supervision used was the same as the one used in the MNIST sums of digits experiments.

**Figure 5:**
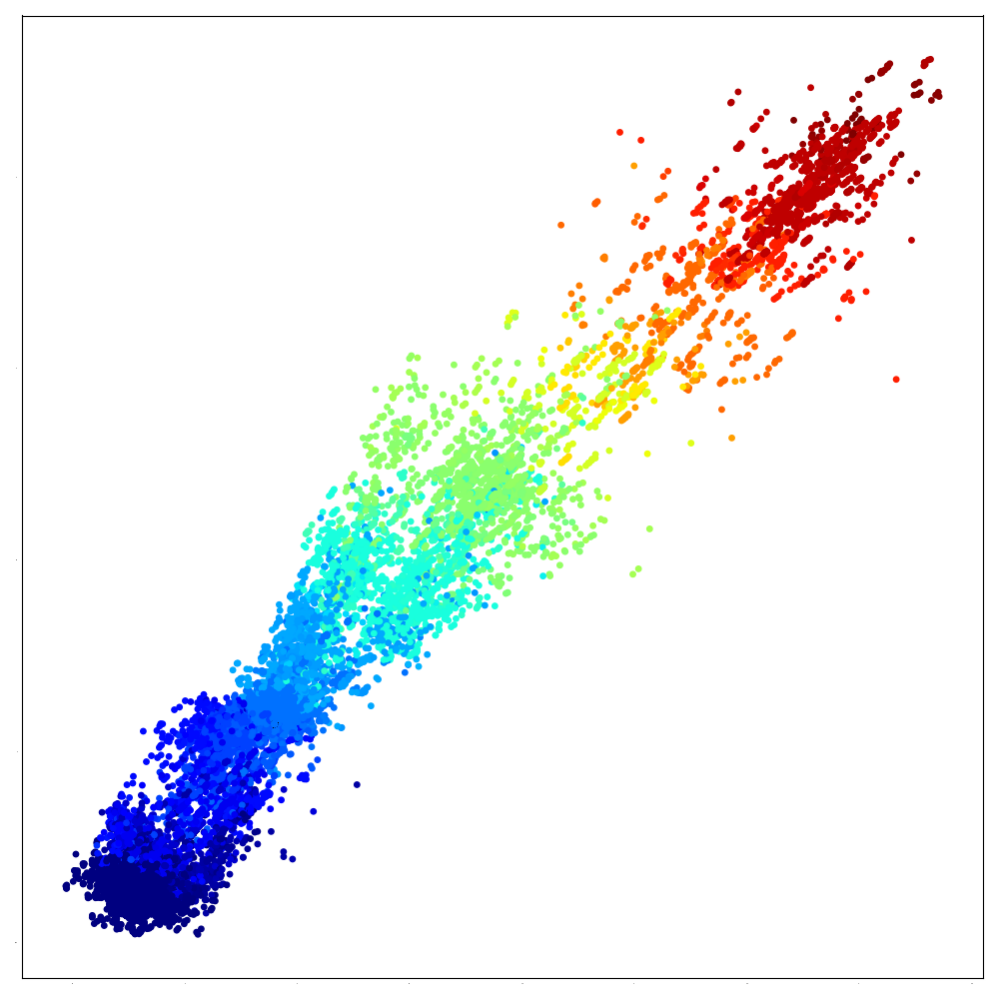
Ordinal embeddings for the Reuters dataset.

**Results.** The results are shown in Figure 5. We embedded each document as a set of paragraphs in a two-dimensional space. As we can notice from the colors that indicate the label of the documents, the clusters are clearly distinguishable. Moreover, we conducted a quantitative evaluation on the obtained embeddings. For this, we computed the centroid of each set from the learned feature vector. Then, we trained an MLP classifier to predict the label associated with the *n* documents. We compared the mean accuracy of our approach to the baseline. We obtained an improvement of 5% in the mean accuracy, which proves that our obtained embedding are better suited for downstream tasks.

## 5. Related Work

In this section, we briefly summarize work that is related to our approach, both in the realm of ordinal embeddings, as well as in finding set representations.

### 5.1. Ordinal Embeddings

In recent years, ordinal data have received a growing interest in machine learning. The ordinal embedding problem has been studied from different points of view, for example: the question of finding the minimum number of triplets necessary to determine an ordinal embedding in the Euclidean space was tackled in [9] and further extended and generalized in [22]. Multiple methods have been designed to deal with triplet similarity. They typically produce representations of data points as low-dimensional Euclidean vectors. In particular, Generalized Nonmetric Multidimensional Scaling (GNMDS) [3] relies on a max-margin approach to minimize a hinge loss. Stochastic Triplet Embedding (STE) [6], on the other hand, assumes a Gaussian noise model and minimizes a logistic noise. The crowd kernel model [5] makes the assumption that triplets have been generated by an explicit noise model. It is worth mentioning that these models adopt a classification scheme to solve the problem by predicting the label of the relative comparisons. Another notable work is [7], which solved the ordinal embedding problem via a reduction to the problem of embedding nearest-neighbor graphs. Moreover, these methods rely on expensive gradient projections and are unsuitable for large datasets. The main purpose of those methods is to facilitate data visualization of similarity inferred from human assessments. However, other tasks employing similarity triplets have been studied, such as medoid estimation [23], density estimation [24], or clustering [25]. Closely related to our approach is [19], which employed deep learning to scale the ordinal problem to large datasets.

### 5.2. Sets’ Representation

Machine learning on sets includes different subgroups depending on the nature of the input and output (e.g., vector-to-set, set-to-set, set-to-sequence). To the best of our knowledge, we are the first to propose an approach for set-valued input that does not rely on features. However, there are different works in the literature that are related to ours, more specifically in the set-to-set domain, where both the input and output are structured as sets. Notable examples are [26], which offered a permutation-invariant function for inference over sets by relying on the summation of all element representations prior to further nonlinear transformations. Other less recent works in the set-to-set domain are [27,28,29]. A more complete comparison of set encoders can be found in the next section. It is important to clearly differentiate our work, which falls into the set-to-set mappings, from some related works on vector-to-set mappings [30,31]. In fact, our work relates more to [26]. The main difference is that the input to our model is necessarily a set of items, albeit without features. Notable works in the vector-to-set literature are suited to tasks such as object detection, taking as input a feature representation of images and producing a set of coordinates for the bounding boxes. Loosely related are also methods such as in [32,33,34] that learn a permutation matrix for sets of items. Once the permutation matrix is learned, it is applied to the input set, hence turning the set into an (ordered) sequence. Once again, our method differs because, there, the output set is not ordered, hence not a sequence.

### 5.3. Sets Encoder Models

The goal of a set encoder is to encode an input set into an embedding vector as an output. Several studies have proposed different approaches for performing this task. In this section, we list the most relevant to our work and provide a more thorough comparison in order to deepen the comparison from the previous section. As stated earlier, in this work, we propose a deep neural architecture for encoding sets without features. Moreover, our approach belongs to the set-to-set category. However, since the ordinal feedback available is only at the set level, the optimization is performed on the embedding of the set rather than the encoding of the single items that compose the set. The most natural comparison to our work is *Deep Sets* [26], which provides a robust mathematical analysis for designing permutation-invariant and permutation-equivariant deep learning models. This framework offers a simplified procedure by relying on the summation of all elements’ representation for obtaining the set feature vector, which is consequently transformed into the desired output (e.g., the class probability for classification or a single number for set regression). Our approach is a generalization of the Deep Sets framework in which we do not require the input feature of the elements of the sets. Additional main differences from our work are that our set representation is a probabilistic measure rather than a point vector and the permutation-equivariant function is the mean rather than the sum.

Other works have tackled the task of finding robust set encoders. They largely differ from our proposition, but we discuss them for the sake of completion. The *Pointer Network* [28] is an encoder-decoder architecture that provides a modified attention mechanism, and its main goal is to learn the target reordering of the input elements. An important characteristic of Pointer Networks is that they do not treat set-valued input in the strict sense. In fact, the input is treated through sequential recurrent neural networks; hence, the obtained representation is not permutation-equivariant. The primary applications of Pointer Networks are tasks where the target output is a reordering or permutation of the elements of the initial input. This reordering is based on pointers to indices of the original input sequence.

Another important set encoder architecture is represented by the *Read-Process-and-Write-Model*. This is a neural network architecture made of different blocks, which aims to obtain a permutation-invariant representation of the input set and learn a mapping to arbitrary target outputs. It relies on an attention mechanism to satisfy the property of permutation-invariance and can be seen as a special case of *Memory Networks* [35]. In fact, it is a recurrent neural network model that creates a memory representation of each element in the input sequence and accesses the representation via the attention mechanism.

Among the most complex methods designed to handle set input problems, there is the *Set Transformer* [36]. The Set Transformer consists of stacked multi-head self-attention layers for both the internal encoder and decoder, as seen in the classic Transformer [37]. One main difference from the previously described set encoding methods is that instead of using a fixed pooling operation such as sum() or average() to ensure permutation-invariance. It employs a parameterized pooling function that is learned and therefore results in being much more adaptive to the particular task at hand. The Set Transformer [36] is designed to model higher-order interactions among elements and their subsets within the input set. Its key advantage is that it concurrently encodes the entire input set through a sequence of permutation-equivariant *Set Attention Blocks* (SABs). By comparison, the previously discussed Deep Sets and our proposed approach method obtained element features independently of other input set elements. The main limitation of the Set Transformer is its computational cost. In fact, the SABs require quadratic complexity $O(n^{2})$ with *n* being the cardinality of the input set. A lower projection was proposed by the authors to try to overcome this limit bringing the overall complexity to O(mn) with *m* being the chosen number of inducing points for the low-rank projection.

Reference [30] proposed a permutation-invariant approach that derives from the the naive method of sorting all the elements of the input set by a chosen feature. However, when the output is a set, this approach leads to discontinuities, which the authors described as the *responsibility problem*. In a nutshell, these discontinuities arise whenever two elements are swapped in the input and the output. To avoid this difficulty, the authors developed a novel pooling method, which sorts each feature across the elements of the input set and then performs a weighted sum. This allows the model to remember the permutation applied through the feature-wise sorting and apply its inverse in the decoder. This process restores the original, arbitrary order of the input elements making the encoding a permutation-equivariant operation, preventing the discontinuity in the outputs of the model.

Another interesting approach to encode sets based on the reordering of the input is the Janossy pooling approach by [38]: the symmetric (permutation-invariant) encoding function is expressed as the average of a mixture of permutation-sensitive functions applied to all re-orderings of the original input. Generating all permutations of a set results in n! intermediate inputs, all of which would then require the application of the chosen permutation-sensitive function. However, this approach is not tractable. To mitigate this, the authors proposed a number of strategies, among them the use of a smaller number of selected canonical orderings that are presumed to carry relevant information.

*PointNet* by [27] is a neural architecture for encoding 3D point clouds. An additional constraint for this architecture is that the output should be independent of the translation or rotation of the input point cloud. Loosely speaking, PointNet first obtains an embedding of each of the input points through stacked, fully connected layers in the form of an MLP, such that each element is identically and independently transformed. This permutation-equivariant representation is then pooled via the max() operator (per dimension) and further transformed through an additional fully connected layer. Finally, the obtained point cloud encoding is concatenated with the embedding of each point. This combination of local and global features is shown to be crucial for point segmentation tasks.

The *AttSets* model, proposed by [39], uses weighted attention to obtain a permutation-invariant representation of the input set. This model was originally meant to be applied to a multi-view 3D reconstruction task, where a set of images of the same object from different angles is used to estimate its true 3D shape. In order to achieve this, each element of the set is individually and independently transformed via a learned attention function, which can take the form of an MLP or a multidimensional CNN, according to the form of the input. The output of this function is normalized via softmax() and then used as an attention mask over the original input elements. This allows the model to learn to pay a varying degree of attention to individual dimensions of the input elements’ representations. Finally, the original input elements are multiplied by the attention mask and summed together to a fixed length set encoding.

Finally, the *RepSet* [40] model consists of stacked feed-forward, fully connected layers, as in the Deep Sets method [28], followed by a custom permutation invariant layer replacing the sum() operator. This layer was inspired by the concepts from the field of bipartite graph matching. The permutation-invariance is achieved through a configurable number of hidden sets (potentially of different sizes), whose elements correspond to columns of trainable weight matrices. These are then compared with the elements of the actual input set to create matrices that are fed the Hungarian algorithm. The resulting values can be further transformed through standard neural network layers according to the problem at hand. One limitation of this approach is the computational complexity of $O(mn+n^{2}\log n)$, where *n* is the cardinality of the input set and *m* is the chosen number of hidden sets.

## 6. Conclusions

We proposed an approach to solve the ordinal embedding problem when the input is under the form of sets of items and the feedback is available only for triplets of sets. Our approach maps the objects in a low-dimensional space endowed with the Wasserstein distance. This is based on learning a representation for sets and taking advantage of the common statistic for sets, which is the centroid. Each set is described by a location parameter, its centroid, and a scale parameter, which represents the spread of the set. We argue that reformulating the problem under this point of view allows prompting fewer triplets comparisons for a greater number of learned items. Our algorithm is suitable when the input sets have variable size. Moreover, a trade-off between the precision of the individual embeddings and the accuracy of the overall density estimation has to be taken into account when choosing the size of the input sets. In a number of experiments on different datasets, we demonstrated the validity of our approach. We showed that the proposed framework is robust and beneficial when the triplet comparisons are limited. Overall, with our proposed approach, we were able to obtain valid embeddings that can be used for downstream tasks. In conclusion, we think that our main idea should be readily extensible to a similar domain, even including features, such as set-to-sequence, set-to-graphs, or set-to-set. Future directions of improvement involve extending the model to consider pairwise or more complex interactions among the elements of a given set. In fact, so far, the encoding step focuses on one element at time. Moreover, an additional way of improvement might be to relax the link between the cardinality of the input set and the precision of the output embedding. Finally, a next improvement we aim to make would be to improve the aggregation function by using a parameterized and learned function, which could improve the performance according to the task at hand.

## Figures and Tables

**Table 1:** Procrustes distance from the ground truth and learned embeddings. Smaller (bold numbers) is better.

	Ours	Baseline
Circles	**0.12**	0.18
Moons	**0.09**	0.33
Spirals	**0.25**	0.91
Blobs	**0.03**	0.04

**Table 2:** Mean accuracy obtained for the classification of single MNIST digit embeddings. Best results are in bold.

	Baseline	Ours
k=10	0.76	**0.78**
k=25	0.76	**0.82**
k=50	0.77	**0.79**

**Table 3:** Pearson correlation coefficient between $l_{2}$ of the different embedding vectors and the Chen score for all possible pairs of poker cards.

Figure	Pearson Coeff.
Figure 4a	0.58
Figure 4b	0.68
Figure 4b	0.65

## Data Availability

No new data were created or analyzed in this study. Data sharing is not applicable to this article.
